# Expanding Sparse Radar Depth Based on Joint Bilateral Filter for Radar-Guided Monocular Depth Estimation

**DOI:** 10.3390/s24061864

**Published:** 2024-03-14

**Authors:** Chen-Chou Lo, Patrick Vandewalle

**Affiliations:** Processing Speech and Images (PSI), Department of Electrical Engineering (ESAT), KU Leuven, 3001 Leuven, Belgium; chenchou.lo@kuleuven.be

**Keywords:** depth estimation, nuScenes, multimodality, radar, signal expansion, joint bilateral filter

## Abstract

Radar data can provide additional depth information for monocular depth estimation. It provides a cost-effective solution and is robust in various weather conditions, particularly when compared with lidar. Given the sparse and limited vertical field of view of radar signals, existing methods employ either a vertical extension of radar points or the training of a preprocessing neural network to extend sparse radar points under lidar supervision. In this work, we present a novel radar expansion technique inspired by the joint bilateral filter, tailored for radar-guided monocular depth estimation. Our approach is motivated by the synergy of spatial and range kernels within the joint bilateral filter. Unlike traditional methods that assign a weighted average of nearby pixels to the current pixel, we expand sparse radar points by calculating a confidence score based on the values of spatial and range kernels. Additionally, we propose the use of a range-aware window size for radar expansion instead of a fixed window size in the image plane. Our proposed method effectively increases the number of radar points from an average of 39 points in a raw radar frame to an average of 100 K points. Notably, the expanded radar exhibits fewer intrinsic errors when compared with raw radar and previous methodologies. To validate our approach, we assess our proposed depth estimation model on the nuScenes dataset. Comparative evaluations with existing radar-guided depth estimation models demonstrate its state-of-the-art performance.

## 1. Introduction

With the rapid advancement of research on autonomous vehicles, various 3D algorithms have emerged to facilitate self-driving, encompassing approaches for depth estimation, 3D object detection, recognition, and segmentation. Among these, accurate outdoor depth estimation algorithms are crucial for improving 3D applications. Accordingly, researchers have proposed numerous monocular and stereo depth estimation algorithms [1,2,3,4,5,6,7,8] that exhibit notable improvements. However, each of these algorithms has its own set of weaknesses: stereo estimation is sensitive to homogeneous surfaces, environmental lighting, and texture conditions, while monocular depth estimation suffers from ill-posed and lack-of-depth characteristics. To address these limitations, approaches incorporating both camera images and lidar data as input have been suggested [9,10,11,12], leveraging the additional depth guidance from lidar to compensate for the limitations of camera features.

Despite lidar’s accurate depth measurements, its high cost and sensitivity to weather conditions limit its usage and performance. As an alternative, some researchers have explored integrating radar as an extra depth guidance into camera-based monocular depth estimation models [13,14,15,16]. While these models have shown promising improvements compared with camera-only models, integrating radar, especially using real data such as the measurements in the nuScenes dataset [17], presents challenges. The radar signal in nuScenes is two-dimensional, with only one beam positioned horizontally at a fixed height in the real world.

**Figure 1:**
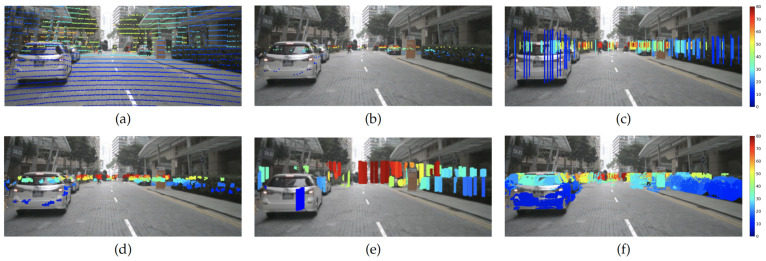
Samples from nuScenes [17] with lidar and different radar formats: (**a**) an image with 1 sweep of sparse lidar projection, (**b**) 5 sweeps of raw sparse radar projection, (**c**) height-extended radar [14], (**d**) $S^{3}$ radar (ad hoc) [18], (**e**) MER with RC-PDA ≥0.5 [15], (**f**) proposed joint bilateral filter expansion. All the point sizes are dilated for better visualization. The color of lidar and radar data indicates the distance, ranging from 0 m (blue) to 80 m (dark red).

Additionally, radar data are extremely sparse compared with lidar or RGB image data, with less than 50 radar points on average for a corresponding 900×1600 image, resulting in a 0.003% density. Projecting radar points onto the corresponding image introduces inaccuracies due to the large width of the radar beam and the location difference between the camera and radar sensor, further complicating the fusion of radar in a depth estimation model. Existing methods employ preprocessing techniques for sparse radar to improve the performance of radar-guided depth estimation models by filtering the noisy measurements [13] or extending the resolution. In an earlier work, we proposed to extend each raw radar point to a vertical line to extend the resolution [14], while other methods offered to train a neural network to enhance the radar data [15,18,19]. However, these existing methods either take only spatial information into consideration or expand with a lidar-supervised neural network. Figure 1 illustrates lidar (a), raw radar (b), preprocessed radar from previous studies [14,15,18] (c–e), and our proposed radar (f). It is evident that our proposed radar offers improved density compared with others while remaining visually consistent with the corresponding image.

In this work, we propose a novel radar expansion method based on the joint bilateral filter to address sparsity and limited vertical view problems. Unlike previous methods that consider only spatial or range difference, our expansion method utilizes an algorithm based on the joint bilateral filter kernel to expand based on spatial and color intensity differences. While [15,18] suggested a lidar-supervised pretrained deep learning model to expand density and remove noisy radar points, our method relies solely on a corresponding camera image as a guidance signal for calculating joint bilateral filter confidence. In the current era of widespread neural network use, rather than relying on lidar-supervised neural networks to improve radar resolution, we are pioneering the adoption of a well-designed and established traditional image filter known as the joint bilateral filter for radar expansion. In contrast with conventional techniques that assign a weighted average of neighboring pixels to the current pixel, our approach expands sparse radar points by computing a confidence score grounded in the values of spatial and range kernels with sparse radar and reference images. Our proposed approach significantly increases sparse radar density while maintaining intrinsic errors within an acceptable range.

The main contributions of this work include the following:

- The proposal suggests employing a joint bilateral filter and calculating a corresponding confidence map to facilitate radar expansion.
- The use of a range-aware window size for expanding radar, providing a better expanding region and higher resolution compared with a fixed window.
- The ability of the proposed expansion method to increase radar points by over 1000 times while minimizing intrinsic errors.
- The flexibility of our method, which does not require lidar supervision during training and can be applied to lidar-free or unsupervised datasets.
- Superior performance for depth estimation compared with previously proposed radar preprocessing methods under the same model settings across various evaluation metrics.

The structure of the paper is as follows: Section 2 reviews related works on lidar depth completion and radar-guided estimation. Section 3 introduces the bilateral filter and our proposed radar expansion method. Section 4 discusses experiments and results, and finally, Section 5 concludes the paper.

## 2. Related Works

In this section, we review representative works in the domains of monocular depth estimation, camera-lidar depth completion, and radar-guided depth estimation.

### 2.1. Monocular Depth Estimation

One of the essential challenges in the task of depth estimation is monocular depth estimation, where a model predicts depth based on monocular images. Early approaches to monocular depth estimation primarily utilized different encoder and decoder structures, regression constraints, or additional information inferred from camera images. Pioneering this field, Saxena et al. [20] proposed a model capturing depths and their relationships using Markov random fields, later extending their work to 3D model generation [21]. Eigen et al. [3,4] introduced a multiscale convolutional neural network to extract local and global features for monocular depth estimation, and since then, numerous works have been proposed, exploring different multiscale architectures [6,22], incorporating additional constraints such as extra semantic information [23] and optical flow [24]. Fu et al. [5] leveraged scene understanding and a ResNet [25] module, also reformulating depth estimation learning as an ordinal regression problem. While most works are based on convolutional backbones, Ranftl et al. [26] proposed a dense prediction transformer that employs a vision transformer [27] as a backbone encoder and decodes features into pixelwise dense depth. However, the ill-posed characteristic, where an infinite number of scenes can map to the same image, imposes limitations on the performance of monocular depth estimation models.

### 2.2. Camera-Lidar Depth Completion

To counter this, several researchers have proposed incorporating lidar as an additional guidance signal to compensate for the lack of depth information. Ma and Karaman [9] proposed concatenating low-resolution lidar with single images in an early-fusion manner to generate a dense output, and further extending the approach to an unsupervised version [10]. Wong et al. [28] employed spatial pyramid pooling to densify sparse lidar and learned topology shapes using synthetic data. Hu et al. [29] utilized a two-branch backbone for image and depth input, and merged their representations. Vangansbeke et al. [30] fused predictions based on confidence maps from both modalities. Li et al. [31] leveraged multiscale structure learning, proposing a model that takes input from different resolutions of sparse lidar, and is supervised by multiscale ground truth lidar. Qiu et al. [32] used surface normals as an intermediate representation. Jaritz et al. [33] learned to predict additional semantic segmentation to enhance depth completion. While lidar proves effective in providing supplementary depth information, its high cost and sensitivity properties create a gap between academic research and practical business usage.

### 2.3. Radar-Guided Monocular Depth Estimation

Due to the lower cost and robustness of radar in comparison with lidar, researchers have begun incorporating sparse radar data into monocular depth estimation models as an alternative source of depth information instead of lidar. However, the inherent characteristics of sparsity and noise in radar pose challenges, prompting most proposed approaches to address these issues before integrating radar data with images. Lin et al. [13] investigated the effects of various fusion approaches based on the sparse-to-dense model [9] and suggested a two-stage prediction method to filter noisy points in raw radar data. Lo and Vandewalle [14] extended raw radar points to a fixed height in real-world coordinates and projected them onto the image plane to address both sparsity and limited view issues. Lee et al. [16] employed a multitask strategy, generating additional 2D object detection and semantic segmentation outputs to enhance depth estimation performance. Long et al. [15] developed a neural network to densify radar depth, guiding depth estimation with additional confidence maps and expanded radar information. Huang et al. [18] enhanced sparse signals based on pixel intensity differences in RGB images and trained a superdensity neural network to address low-density and imbalanced distribution issues.

Singh et al. [19] introduced RadarNet, which initially maps an arbitrary number of radar points to object surfaces in an image, serving as the first stage to generate a quasi-dense radar depth. This is achieved through radar-camera correspondence from a single image and radar point cloud. Additionally, they proposed FusionNet, utilizing a gated fusion network as the second stage, to regulate the fusion of multimodal features and to estimate the final dense depth. Lo and Vandewalle [34] proposed a dedicated transformer module for radar input, reassembling features from radar and images instead of using readout tokens, as in [26,27].

These studies indicate that integrating radar data as additional depth guidance can improve the performance of depth estimation models. However, the existing methods face challenges. One issue is that prior efforts tend to concentrate solely on spatial information or require training with lidar, limiting their effectiveness. While expanding radar based on spatial information offers only a limited improvement in resolution, a lidar-supervised expansion network shows improved radar depth but lacks flexibility for adaptation to datasets without lidar information. Another issue stems from the inherent drawbacks of raw radar data, specifically their sparsity and limited vertical view characteristics, necessitating preprocessing. This preprocessing step becomes crucial for optimal performance in radar-guided depth estimation models. Our proposed solution is to expand sparse radar using both spatial and range information from its reference image, recognizing the fundamental correlation between camera images and radar data in autonomous vehicle datasets.

## 3. Methodology

**Figure 2:**
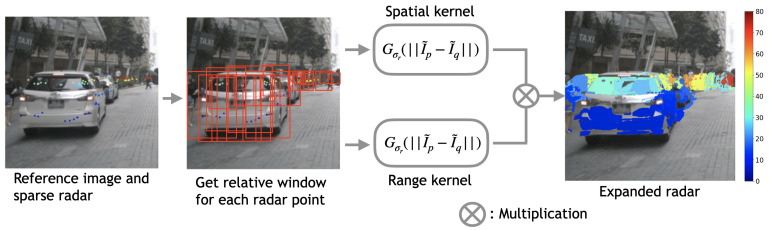
Illustration of the proposed joint bilateral filter expansion process. The expansion window for each radar point is initially determined by a predefined width and height, alongside the distance of the radar point under consideration, highlighted with red frames. Subsequently, both spatial and range kernels are employed to determine the expansion confidence score for every point within the window. The final radar expansion is determined by considering the bilateral confidence alongside a predefined threshold. The details of the proposed joint bilateral expansion method are summarized in Algorithm 1. The color of radar data indicates the distance, ranging from 0 m (blue) to 80 m (dark red).

Our objective is to develop a radar expansion methodology aimed at attaining an enhanced radar format and optimizing the performance of existing radar-guided depth estimation models. To address the inherent sparsity and limited field of view in radar data, we propose expanding the raw radar data, making use of the concept of a joint bilateral filter (JBF). The rationale is that radar data and images share the same object information but in different modalities. Additionally, the joint bilateral filter is a filter designed to enhance the resolution of a target image by utilizing information from a related reference image. Thus, we use images as the reference for expansion guidance since they offer abundant color intensity information about objects and surroundings. Instead of directly applying the joint bilateral filter, we propose computing a confidence map by integrating color information from a reference image and taking into account the Euclidean pixel distance in radar. Subsequently, radar expansion is performed based on the confidence scores obtained from the confidence map. Figure 2 depicts the procedural steps of our proposed joint bilateral filter radar expansion method. In this section, we initially introduce the bilateral filter and subsequently offer a comprehensive explanation of our proposed method, encompassing the computation of the confidence map and radar expansion.
**Algorithm 1:** Proposed Joint Bilateral Expansion.***function*** GET_REL_SIZE (d,w,h)     $u\leftarrow (w\times f_{u})/d$     $v\leftarrow (h\times f_{v})/d$     **return** u,v 
**for** each point (x,y,d) in RADAR     Start from point with a larger *d*     u,v← GET_REL_SIZE (d,w,h)     **for** *i* in **range**(−u/2,u/2)          **for** *j* in **range**(−v/2,v/2)               $D_{s}\leftarrow ||\left(x,y\right)-\left(x+i,y+j\right)||$               $D_{r}\leftarrow \left|I\left(x,y\right)-I\left(x+i,y+j\right)\right|$               $C_{JBF}\leftarrow G_{\sigma_{s}}\left(D_{s}\right)G_{\sigma_{r}}\left(D_{r}\right)$ as in (5)               **if** $C_{JBF}\geq \mathrm{Threshold}$ **do**                    RADAR(x+i,y+j) ←RADAR(x,y)**return** RADAR

### 3.1. Joint Bilateral Filter

The bilateral filter is a filter designed to preserve edges while effectively smoothing noise in images. Originally introduced by Tomasi and Manduchi [35], it has become a well-established technique in image processing [36]. The bilateral filter consists of a spatial kernel and a range kernel, taking into account differences not only in spatial distance but also in color values among neighbors. The main concept is that, for a pixel to influence its neighbors, it should be close and have similar values. The bilateral filter is defined as follows:

(1) $$BF{\left[I\right]}_{p}=\frac{1}{W_{p}}\sum_{q\in S}G_{\sigma_{s}}(||p-q||)G_{\sigma_{r}}\left(\right|I_{p}-I_{q}\left|\right)I_{q},$$

where $G_{\sigma }$ denotes the 2D Gaussian kernel:

(2) $$G_{\sigma }\left(x\right)=\frac{1}{2\pi \sigma^{2}}exp\left(-\frac{x^{2}}{2\sigma^{2}}\right).$$

$G_{\sigma_{s}}$ and $G_{\sigma_{r}}$ are the spatial and range kernels, respectively, and $\sigma_{s}$ and $\sigma_{r}$ will specify the amount of filtering for the image *I*. $I_{p}$ is the image value at pixel position *p*. The kernels are calculated against all possible image locations *q* in the set of window size *S*. ||p−q|| refers to the Euclidean distance between pixel locations *p* and *q*, and $|I_{p}-I_{q}|$ is the absolute difference in color between pixel locations *p* and *q*. $W_{p}$ is a normalizing factor that ensures pixel weights sum to 1.0:

(3) $$W_{p}=\sum_{q\in S}G_{\sigma_{s}}(||p-q||)G_{\sigma_{r}}\left(\right|I_{p}-I_{q}\left|\right).$$

Note that the spatial and range kernels in the bilateral filter are multiplied, showing that both elements matter and that no smoothing occurs if either is close to zero.

With the edge-preserving property while smoothing, several works have introduced the joint bilateral filter that applies a second guidance image in the range filter to perform upsampling [37], depth reconstruction [38], and data fusion [39]. The joint bilateral filter is defined by the following:

(4) $$JBF{\left[I\right]}_{p}=\frac{1}{W_{p}}\sum_{q\in S}G_{\sigma_{s}}(||p-q||)G_{\sigma_{r}}\left(\right|{\tilde{I}}_{p}-{\tilde{I}}_{q}\left|\right)I_{q},$$

where $\tilde{I}$ is the guidance image.

### 3.2. Proposed Expansion Method

We propose expanding the sparse raw radar using the joint bilateral filter, leveraging both spatial and range kernels. However, in previous upsampling works employing the joint bilateral filter, the source was dense but with some missing pixels that needed to be filled. In our scenario, we contend with an extremely sparse source radar depth and a monocular corresponding reference image. Additionally, the bilateral filter traditionally involves a weighted average of neighboring pixels. Given our sparse source and the goal of expansion, calculating a weighted average based on neighboring pixels and assigning it to a specific pixel is not applicable. Instead, we perform expansion based on the confidence of how each radar point can contribute to its nearby pixel locations. Consequently, we calculate the JBF confidence $C_{JBF,p}$ of nearby points for every radar point *q* against given centered raw radar points *p* with both spatial and range kernels in window *S*, as follows:

(5) $$\begin{array}{c} C_{JBF,p}=G_{\sigma_{s}}(||p-q||)G_{\sigma_{r}}\left(\right|{\tilde{I}}_{p}-{\tilde{I}}_{q}\left|\right),\;\mathrm{for}q\in S. \end{array}$$

Algorithm 1 summarizes our proposed joint bilateral expansion method. The window size has to be defined before calculating the bilateral confidence. Instead of employing a fixed window size in the image plane as in [15], we opt for a fixed size in the real 3D world, which is an adaptive size in the image. Consequently, we initially project the real-world size onto the relative pixel size in the image plane based on the depth of a given point and the camera’s intrinsic value. The idea is that a closer radar point should have a larger window for expansion, while a more distant point should have a smaller window.

**Figure 3:**
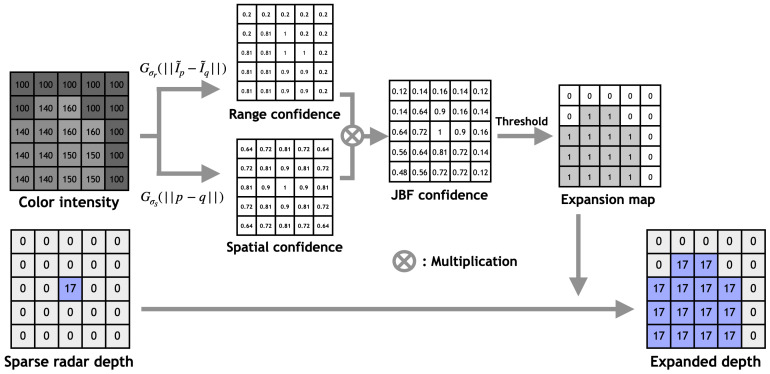
Schematic diagram illustrating the proposed radar expansion method. The sparse radar depth and color intensity from camera images are given features. Following the computation of the expansion window for each sparse radar point, range and spatial confidence maps are calculated based on color and distance differences. The JBF confidence map is obtained by multiplying the range and spatial confidence maps, and the expansion map is generated after applying a threshold on the JBF confidence map. Finally, the expanded radar depth is obtained by combining the raw sparse radar depth with the expansion map.

**Figure 4:**
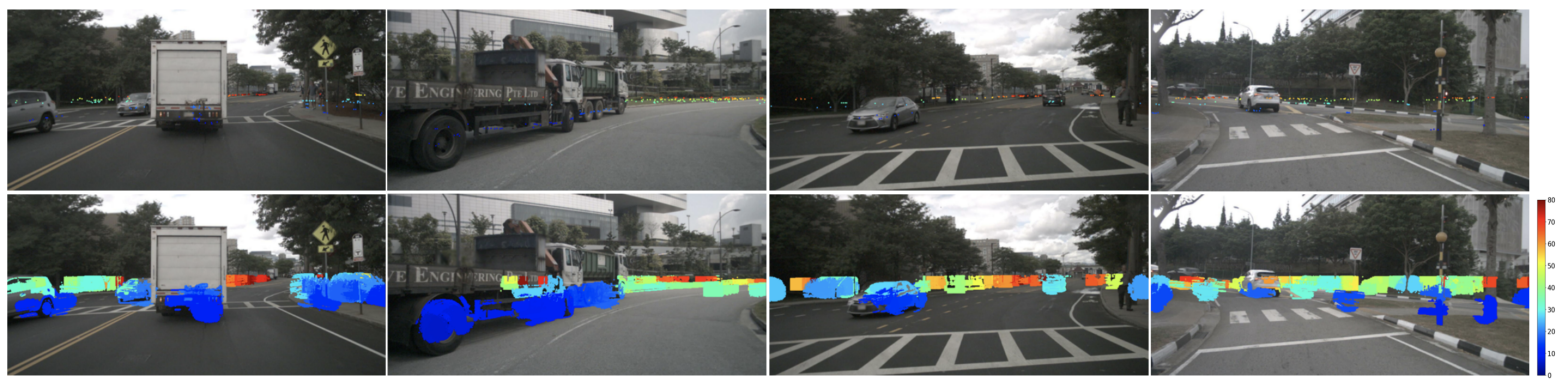
Samples of the proposed radar expansion. **Top** row: RGB image with the 5-frame raw radar. **Bottom** row: RGB image with the proposed JBF radar with $\sigma_{s}=25$ and $\sigma_{r}=10$. All the point sizes are dilated for better visualization and better viewing in color. The color of expanded radar indicates the distance, ranging from 0 m (blue) to 80 m (dark red).

Subsequently, we compute the bilateral confidence of nearby points for every point in the sparse raw radar. Finally, we assign the depth of the current point to the nearby point if its joint bilateral confidence is equal to or larger than a predefined threshold. Figure 3 outlines the computation process of the JBF confidence map and the subsequent generation of an expanded radar depth based on the expansion map and sparse radar depth. Figure 4 shows samples of the raw radar and the proposed JBF expanded radar. It is clear that, based on both spatial and range constraints, our proposed method effectively captures the shape of objects.

### 3.3. Intrinsic Error

To show the performance of our proposed expansion method, we evaluate its intrinsic error by comparing the expanded radar against the ground truth sparse lidar. Error metrics are exclusively computed at locations where both lidar and radar data are available. The result of intrinsic error comparison with existing expansion methods is depicted in Table 1. Our proposed method outperforms all the other methods on density in that the number of points is expanded from an average of 39 points (0.01%) in raw radar to an average of 103,249 (28.68%) points per radar depth map. Since MER [15] is an expansion method that is supervised by ground truth lidar, it has the ability to cancel noisy measurements. As a result, JBF has lower errors compared with raw radar, height-extend radar [14], and $S^{3}$ radar [18] on δ and RMSE, but errors on these metrics are slightly higher compared with MER. It is noteworthy that the MER method necessitates the incorporation of both image and lidar data during its training process. In contrast, our proposed approach eliminates the need for training with lidar data, thereby facilitating its adaptability to other self-driving datasets.

## 4. Experiments

### 4.1. Implementation Details

**NuScenes Dataset.** We use the nuScenes dataset [17], consisting of 6 cameras, 5 FMCW radars, and a 32-beam Velodyne lidar, for conducting our experiments. The nuScenes dataset is currently one of the most comprehensive multimodal autonomous driving datasets, and most previous radar-guided depth estimation models are also based on this dataset. It consists of 1000 driving scenes captured in Boston and Singapore, and each scene contains roughly 40 manually synchronized key-frame samples from a 20 s recording of outdoor driving. The 1000 scenes are officially split into 700 training, 150 validation, and 150 testing scenes. We use the front-view data only among all views and train and test on training and validation samples with the same setting as in previous works.

**Models Used in Experiments.** Our aim is to propose a novel radar expansion method, so we directly use models from previous works [13,14,34], but the radar is replaced with our proposed radar. All the experiments are implemented in PyTorch [40] and trained on a Tesla V100 GPU on a DGX-1 server. The models are taken from the code released from the original papers, and we closely follow the same training and evaluation setting. To ease computation, the camera images, projected lidar depth, and radar depth are downsampled from the original shape of 900×1600 to a smaller shape. For radar-guided depth estimation experiments, we conduct experiments on DORN [14] and RCDPT [34]. The weights of ResNet-101 [25] in DORN are initialized via the pretrained model on ILSVRC [41,42]. The RGB images, lidar depth, and radar depth are downsampled from the original size of 900×1600 to 450×800.

As the sky region contributes no depth values, the RGB images, lidar, and radar depth are further cropped into a shape of 350×800 as the training resolution. In RCDPT, both input camera images and radar depth are cropped into a size of 384×384. For radar inference experiments, the input image is resized to 450×800 and also further cropped into a shape of 350×800. For all experiments, a polynomial decay with a starting learning rate of 0.0001 and a power rate of 0.9 as the learning strategy is applied in the training phase. The batch size is set to 4, and momentum and weight decay are set to 0.9 and 0.0005, respectively. While training, we further use data augmentation for RGB images to improve the robustness as follows: gamma contrast in range (0.9, 1.1), brightness adjustment in range (0.9, 1.1), color adjustment in range (0.9, 1.1), and horizontal flipping with 0.5 probability. We train DORN, RCDPT, and S2D for 30 epochs on the nuScenes official training splits and test on the nuScenes official validation splits. The evaluation metrics used are the standard evaluation metrics also used in previous works, and calculations for all experiments are based on the size of 350×800 using ground truth sparse lidar with a maximum distance of 80 m. Note that the evaluation metrics are only calculated at the pixel locations with valid points in the ground truth sparse lidar. For radar expansion in our experiments, we set $\sigma_{s}=25$, $\sigma_{r}=10$, and the threshold is 0.05. We use a shape of 2.35 m × 2.35 m for the fixed expansion size in the real world, which is the average shape of vehicles from the released information of objects in the nuScenes dataset.

### 4.2. Evaluation Metrics

We use the standard metrics as in previous works to evaluate our results.

**Threshold Accuracy (**$\delta_{n}$**):** % of *Y* s.t.

$$\begin{array}{c} max\left(\frac{\hat{Y}}{Y},\frac{Y}{\hat{Y}}\right)=\delta_{n}<1.25^{n} \end{array}$$

**Root Mean Square Error (RMSE):**

$$\begin{array}{c} \sqrt{\frac{1}{N}\sum_{i=1}^{N}{∥Y-\hat{Y}∥}_{2}^{2}} \end{array}$$

**Absolute Relative Error (AbsRel):**

$$\begin{array}{c} \frac{1}{N}\sum_{i=1}^{N}\frac{\left|Y-\hat{Y}\right|}{\left|Y\right|}, \end{array}$$

where *i* is pixels and *N* is the total number of pixels. *Y* and $\hat{Y}$ are the dense prediction and the target depth, respectively.

### 4.3. Radar-Guided Depth Estimation

**Figure 5:**
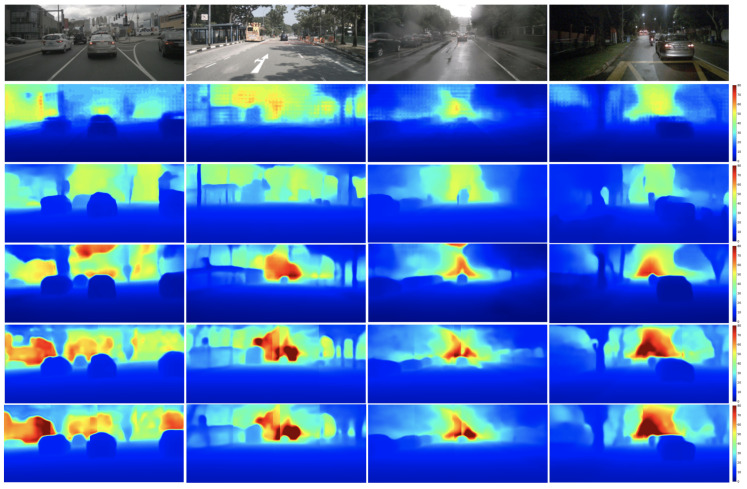
Qualitative comparison of results for radar-guided depth estimation experiments. From top to bottom: input monocular image, DORN_*radar*_ [14], RC-PDA [15], Lin [13], RCDPT [34], our proposed radar with RCDPT. The color of the estimated depth indicates the distance, ranging from 0 m (blue) to 80 m (dark red).

We train our proposed JBF radar with models from DORN_*radar*_ [14] and RCDPT [34], for they are two of the state-of-the-art radar-guided depth estimation models. The quantitative results of the proposed radar and previous works on the nuScenes dataset with a depth range < 80 m are summarized in Table 2. We refer the interested readers to [13,14,15,18,19] for details of raw radar, height-extended radar, MER, $S^{3}$, and RadarNet, respectively. In the bottom two rows of Table 2, we can see that our proposed radar with both DORN_*radar*_ and RCDPT outperforms existing models with different radar formats in all evaluation metrics. It also shows that our proposed radar with the RCDPT model has better performance compared with DORN_*radar*_, which confirms the conclusion in previous works that the transformer backbone can yield better performance than the CNN backbone [26,34]. When comparing identical models trained with different radar formats, namely, DORN_*radar*_ with height-extend versus JBF, and RCDPT with MER versus JBF, the results consistently indicate that the models exhibit better performance when trained with our proposed JBF radar. Figure 5 shows the qualitative results of our proposed radar compared with previous works. Although our proposed radar with RCDPT has better quantitative results, it only provides a little significant improvement in the qualitative results compared with previous models. Some structures can be captured slightly better with our proposed radar, but the results are comparable to [13,34] for the overall perceptions.

### 4.4. Radar Inference Experiments

We further conduct radar inference experiments as in [43] with our proposed JBF radar. The radar inference experiments aim to examine if a model can predict surroundings to a fair extent with only radar input and under lidar supervision during training. The models used in this experiment are DORN_*radar*_ and S2D_*radar*_, and the results of the comparison of our proposed radar and previous methods are shown in Table 3. It is clear that our proposed radar outperforms MER in all metrics on DORN_*radar*_, while it is comparable on S2D_*radar*_. However, MER has higher δ and lower RMSE in the intrinsic error in Table 1 and is generated from a lidar-supervised model. It is fair for our proposed radar to have a comparable performance in one model and a better performance in another model. This also indicates that the intrinsic depth information that radar can contribute is indeed increased through our proposed expansion method comparing the results with the raw radar.

### 4.5. Selection of Spatial and Range Sigma

**Figure 6:**
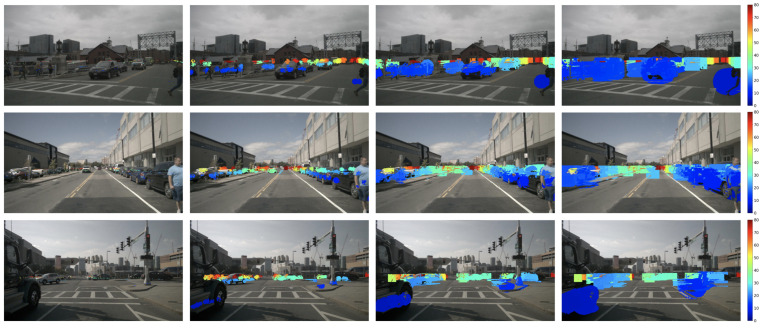
Samples of the proposed radar expansion with different $\sigma_{s}$ and $\sigma_{r}$ in spatial and range kernels. The columns from left to right show the RGB image with 5-frame raw radar. The proposed JBF radar with $\sigma_{s}=10$ and $\sigma_{r}=5$; $\sigma_{s}=25$ and $\sigma_{r}=10$; $\sigma_{s}=50$ and $\sigma_{r}=20$. All the point sizes are dilated for better visualization and better viewing in color. The color of radar data indicates the distance, ranging from 0 m (blue) to 80 m (dark red).

As the Gaussian kernel plays a fundamental role in our proposed method, both spatial sigma and range sigma in the joint bilateral filter significantly influence the expanded radar depth. The function of sigma in the Gaussian filter is to regulate the variation in the kernel: a larger sigma allows more variance, while a smaller sigma restricts variance. To understand the impact of different spatial and range sigma pairs on our proposed expansion method, we trained the RCDPT model under sparse lidar supervision with varying JBF expanded radar depths. Table 4 presents the evaluated results for spatial and range sigma pairs of (10, 5), (25, 10), and (50, 20), and the corresponding expanded results are depicted in Figure 6. In Figure 6, for the sigma pair ($\sigma_{s}$, $\sigma_{r}$) = (10, 5), the expansion is limited due to the smaller sigma values. Conversely, for the sigma pair ($\sigma_{s}$, $\sigma_{r}$) = (50, 20), it is evident that the sigma values are too large, causing many expanded radar points to extend beyond the objects around the original raw radar points. Therefore, we have selected the sigma pair ($\sigma_{s}$, $\sigma_{r}$) = (25, 10), as shown in the third column of Figure 6, which provides a balanced expansion of the raw radar to a fair extent. The evaluated results in Table 4 also confirm that the sigma pair ($\sigma_{s}$, $\sigma_{r}$) = (25, 10) yields the best performance among all three settings. Although the pair ($\sigma_{s}$, $\sigma_{r}$) = (50, 20) results in significantly more expanded points, these points introduce more noise, which leads to the worst performance as indicated in Table 4.

### 4.6. Effects of Employing Only a Single Kernel

**Figure 7:**
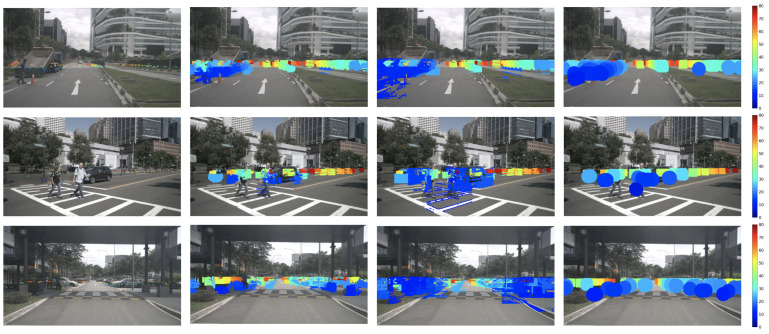
Samples of expanded radar by either a single kernel or both kernels. The columns from left to right show the RGB image with 5-frame raw radar, the proposed JBF radar with $\sigma_{s}=10$ and $\sigma_{r}=5$, range kernel only with $\sigma_{r}=10$, and spatial kernel only with $\sigma_{s}=25$. All the point sizes are dilated for better visualization and better viewing in color. The color of radar data indicates the distance, ranging from 0 m (blue) to 80 m (dark red).

Instead of utilizing both range and spatial kernels as in the joint bilateral filter, we further conduct experiments of expanding the raw radar data using either a single range kernel or a spatial kernel. This experiment aims to demonstrate the effectiveness of the JBF kernels. We set $\sigma_{s}$ to 25 for the spatial kernel expansion, $\sigma_{r}$ is set to 10 for range kernel expansion, and we use ($\sigma_{s}$, $\sigma_{r}$) = (25, 10) for expansion using both kernels. In Figure 7, the radar expanded with the range kernel considers only the differences in color intensity in the reference image, lacking spatial distance information, leading to overexpansion on objects. Conversely, the radar expanded with the spatial kernel takes only spatial distance into account, resulting in circular artifacts due to the absence of color information. The intrinsic error evaluated in Table 5 also reflects the same trend, indicating that the JBF-expanded radar exhibits lower errors compared with methods using a single kernel.

### 4.7. Impact of Using Additional Confidence Map

The JBF confidence map represents the degree of confidence for a given raw radar point to expand to all pixel locations within the expanding window. The expansion process is determined by the JBF confidence map and a threshold, as illustrated in Figure 3. Since the confidence map provides additional information on the expanded radar depth, we conducted an experiment by training the RCDPT model with our proposed JBF radar and confidence map as additional input modalities. Table 6 presents the evaluated results, comparing the performance with and without the use of the JBF confidence map as an additional input feature. The results indicate that a radar-guided depth estimation model, such as RCDPT, can fairly benefit from the inclusion of the confidence map. This additional information helps guide the model’s output more effectively for the expanded radar depth.

## 5. Conclusions

Compared with camera-lidar depth completion, radar-guided monocular depth estimation introduces several challenges related to the intrinsic properties of radar. Our proposed method draws inspiration from the combination of spatial and range kernels in the joint bilateral filter. We adapted the joint bilateral filter and proposed computing a confidence map based on spatial and range differences, followed by the subsequent expansion of radar data. Unlike preprocessing methods in previous works that solely use spatial information or require lidar training, our approach incorporates both spatial and range information. By utilizing only a reference image as prior information, our proposed method can be easily adapted to any other autonomous driving dataset. The experimental results demonstrate that our proposed radar method outperforms existing works across various evaluation metrics. Areas for future work include exploring more effective utilization of the confidence map in a well-designed depth estimation model with a dedicated encoder for specialized information extraction. Additionally, considering iterative applications of the bilateral filter could expand more points while maintaining low intrinsic error. In our current setting, $\sigma_{s}$, $\sigma_{r}$, and the threshold are fixed. However, dynamically determining these parameters based on the distance of the given point could lead to a better-expanded radar depth. The most notable issue in the bilateral filter is its complexity, which is $O({\left|S\right|}^{2})$. Therefore, implementing a faster version is necessary to alleviate computational costs and reduce latency. Moreover, our proposed JBF radar demonstrates an improved resolution for objects and road features, surpassing the capabilities of raw radar data and previous methods using a deterministic and efficient expansion method that does not require training. Consequently, it can be employed in various autonomous driving tasks, such as 3D object detection and path planning, showing an even wider application domain than our initial purpose of depth estimation.

## Figures and Tables

**Table 1:** Intrinsic errors comparing our proposed expansion with state of the art on the nuScenes dataset. Errors are calculated between each radar format and ground truth sparse lidar at the locations where radar and lidar have values. Note that the depth map shape is 450×800, and the maximum evaluated distance is 80 m. The symbols ↑ and ↓ indicate that higher and lower values are better for the metrics, respectively, where the “# points” refers to the number of points in the depth map. Numbers in bold denote the best result.

Method	Lidar	$\delta_{1}$ ↑	$\delta_{2}$ ↑	RMSE ↓	# Points ↑	Density (%) ↑
Raw	-	0.41	0.61	29.93	39.01	0.01
Height-extend [14]	-	0.46	0.67	21.52	9187	2.55
$S^{3}$ (ad hoc) [18]	-	0.43	0.63	28.04	6518	1.81
MER [15]	✓	**0.73**	**0.85**	**11.29**	25,370	7.05
JBF	-	0.59	0.77	14.64	**103,249**	**28.68**

**Table 2:** Quantitative results comparing the proposed method with existing models on the nuScenes dataset. Top rows: baseline models using monocular images only. Middle rows: existing radar-guided depth estimation methods. Bottom rows: DORN_*radar*_ and RCDPT trained from scratch with our proposed JBF radar. The maximum evaluation distance is 80 m. The symbols ↑ and ↓ indicate that higher and lower values are better for the metrics, respectively. Numbers in bold indicate the best result.

Method	Radar Format	$\delta_{1}$↑	$\delta_{2}$↑	$\delta_{3}$↑	RMSE ↓	AbsRel ↓
DORN [5]	-	0.872	0.952	0.978	5.382	0.117
S2D [9]	-	0.862	0.948	0.976	5.613	0.126
DPT [26]	-	0.886	0.957	0.980	5.244	0.106
S2D [9]	Raw	0.876	0.949	0.974	5.628	0.115
DORN_*radar*_ (1-stage) [14]	Height-extend	0.889	0.961	0.984	5.191	0.109
DORN_*radar*_ (2-stage) [14]	Height-extend	0.890	0.960	0.983	5.260	0.108
Lin (1-stage) [13]	Raw	0.884	0.953	0.977	5.409	0.112
Lin (2-stage) [13]	Raw	0.901	0.958	0.978	5.180	0.100
Lee [16]	Raw	0.895	0.958	0.978	5.209	0.104
$S^{3}$ [18]	$S^{3}$	0.798	0.921	0.962	6.77	0.161
$S^{3}$ (GDC) [18]	$S^{3}$	0.799	0.921	0.962	6.76	0.160
FusionNet [19]	RadarNet	0.87	0.95	0.98	5.79	0.12
RC-PDA [15]	MER	0.830	0.917	0.956	6.942	0.128
DPT-Early [34]	MER	0.892	0.956	0.978	5.401	0.099
DPT-Late [34]	MER	0.888	0.958	0.981	5.207	0.104
RCDPT [34]	MER	0.901	0.961	0.981	5.165	0.095
DORN_*radar*_ (1-stage) [14]	JBF	0.901	0.962	0.981	5.228	0.104
RCDPT [34]	JBF	**0.909**	**0.964**	**0.985**	**4.873**	**0.089**

**Table 3:** Evaluation results for radar inference experiments with different methods and input radar. Note that this experiment uses the ground truth sparse lidar as the supervision signal. We used radar with RC-PDA ≥0.5 in MER. CAP refers to the maximum depth range in meters. The symbols ↑ and ↓ indicate that higher and lower values are better for the metrics, respectively. The bold formatting used for numbers denotes the best result.

Model	Input Radar	CAP (m)	$\delta_{1}$↑	$\delta_{2}$↑	RMSE ↓	AbsRel ↓
	Raw radar	80	0.716	0.774	7.817	0.260
Lo [14]	Height-extend [14]	80	0.763	0.844	**6.582**	0.232
	MER [15]	80	0.736	**0.902**	7.781	0.227
	JBF	80	**0.786**	**0.902**	7.684	**0.196**
	Raw radar	80	0.714	0.768	8.151	0.247
Lin [13]	Height-extend [14]	80	0.783	0.865	**6.404**	0.220
	MER [15]	80	**0.801**	0.890	7.290	**0.155**
	JBF	80	0.785	**0.901**	7.698	0.179

**Table 4:** Selection of spatial sigma ($\sigma_{s}$) and range sigma ($\sigma_{r}$) in our proposed expansion method. The symbols ↑ and ↓ indicate that higher and lower values are better for the metrics respectively. The bold formatting used for numbers denotes the best result.

($\sigma_{s}$, $\sigma_{r}$)	$\delta_{1}$↑	$\delta_{2}$↑	$\delta_{3}$↑	RMSE ↓	AbsRel ↓
(10, 5)	0.901	0.961	0.981	5.175	0.093
**(25, 10)**	**0.909**	**0.964**	**0.985**	**4.873**	**0.089**
(50, 20)	0.891	0.959	0.980	5.317	0.102

**Table 5:** Intrinsic errors comparing using either a single kernel or both kernels. Errors are calculated between each radar format and ground truth sparse lidar at the locations where radar and lidar have values. Note that the expansion threshold is 0.05, the depth map shape is 450×800, and the maximum evaluated distance is 80 m. The symbols ↑ and ↓ indicate that higher and lower values are better for the metrics, respectively whereas “# points” refers to the number of points in the depth map. Numbers in bold highlight the best result.

Kernel	($\sigma_{s}$, $\sigma_{r}$)	$\delta_{1}$↑	$\delta_{2}$↑	RMSE ↓	# Points ↑	Density (%) ↑
JBF	(25, 10)	**0.59**	**0.77**	**14.64**	103,249	28.68
Range	(-, 10)	0.54	0.69	19.62	**181,609**	**50.44**
Spatial	(25, -)	0.52	0.66	22.18	166,668	46.29

**Table 6:** The impact of using the confidence map in the RCDPT model. The symbols ↑ and ↓ indicate that higher and lower values are better for the metrics, respectively. The bold formatting used for numbers denotes the best result.

Confidence Map	$\delta_{1}$↑	$\delta_{2}$↑	$\delta_{3}$↑	RMSE ↓	AbsRel ↓
No	0.909	0.964	0.985	4.873	0.089
Yes	**0.911**	**0.967**	**0.986**	**4.735**	**0.087**

## Data Availability

Data are contained within the article. The autonomous vehicle dataset is available on the official nuscenes website (https://www.nuscenes.org/).
